# On‐feet isometric bracing maintains cerebral arterial blood velocity during lower body negative pressure via preload augmentation

**DOI:** 10.1113/EP093648

**Published:** 2026-02-04

**Authors:** Jun Sugawara, Marina Fukuie, Tsubasa Tomoto, Takashi Tarumi, Ai Hirasawa, Shigeki Shibata

**Affiliations:** ^1^ Integrated Research Center for Self‐Care Technology Ibaraki Japan; ^2^ Human Informatics and Interaction Research Institute National Institute of Advanced Industrial Science and Technology (AIST) Ibaraki Japan; ^3^ Faculty of Health Science Kyorin University Tokyo Japan

**Keywords:** lower body negative pressure, orthostatic intolerance, transcranial Doppler, venous return

## Abstract

Orthostatic stress reduces venous return and stroke volume (SV), risking cerebral hypoperfusion despite autonomic compensation. Although lower‐limb counterpressure manoeuvres improve cerebral perfusion in upright posture, their effects on cerebral blood velocity (CBV) during lower‐body negative pressure (LBNP) and the associated mechanisms are not fully defined. We therefore tested whether isometric lower‐limb contraction is associated with preservation of CBV during LBNP, accompanied by attenuated effects of preload reduction. Thirteen healthy young adults (age: 25 ± 5 years; 5 women) completed randomized trials under two conditions: off‐feet (saddle support, relaxed legs) and on‐feet (isometric bracing against a footplate with slight knee flexion). Each condition included 6 min exposures to −30 and −50 mmHg. Systemic vascular conductance declined with increasing LBNP, whereas mean arterial pressure (MAP) was maintained in both conditions. At −50 mmHg, CBV decreased off‐feet but was preserved on‐feet; SV fell less and the compensatory rise in heart rate (HR) was attenuated on‐feet. Repeated‐measure correlations showed that CBV tracked SV (*r*_rm_ = 0.388, *P* = 0.002) and end‐tidal CO_2_ (*r*_rm_ = 0.318, *P* = 0.012), was inversely related to HR (*r*_rm_ = −0.448, *P* = 0.001) and was unrelated to MAP (*r*_rm_ = −0.003, *P* = 0.980) or systemic vascular conductance (*r*_rm_ = 0.193, *P* = 0.129). Thus, isometric lower‐limb engagement is associated with preservation of CBV during LBNP, in a manner consistent with preload‐mediated effects rather than augmented peripheral vasoconstriction. These findings are consistent with proposed mechanisms underlying physical counterpressure manoeuvres and support simple lower‐limb isometric actions to improve orthostatic tolerance.

## Abstract (highlights)

- **What is the central question of this study?**
  Does lower‐limb isometric engagement during lower‐body negative pressure attenuate reductions in cerebral blood velocity (CBV) independently of changes in arterial pressure, and are such effects consistent with preload‐mediated mechanisms rather than reflex tachycardia or vasoconstriction?
- **What is the main finding and its importance?**
  CBV was preserved during on‐feet isometric bracing despite maintained arterial pressure, whereas CBV declined off‐feet. CBV covaried with stroke volume but not with mean arterial pressure, indicating a preload‐related contribution. These results highlight the importance of muscle engagement in stabilizing cerebral haemodynamics during central hypovolaemia and provide physiological insight into counter‐pressure manoeuvres.

## 1 INTRODUCTION

Central hypovolaemia, whether induced by orthostatic stress, haemorrhage or simulated conditions such as lower body negative pressure (LBNP), challenges the maintenance of cerebral perfusion. In humans, tolerance to such stress depends on preserving stable cerebral blood flow through rapid integration of systemic cardiovascular control and intrinsic cerebrovascular autoregulation (Claassen et al., 2021; Schondorf et al., 2001; Stewart, 2012). Although autoregulation buffers cerebral blood flow over a broad arterial pressure range, cerebral blood flow is not pressure‐independent: cardiac output (CO) and arterial carbon dioxide tension exert measurable influences on cerebral blood velocity (CBV) and flow (Ogoh et al., 2022; Willie et al., 2014). Conceptually, systemic haemodynamics reflect the interaction between cardiac factors (stroke volume [SV] and heart rate [HR]) and vascular resistance or conductance (Rowell, 1993).

LBNP is a widely used experimental model of central hypovolaemia that elicits graded reductions in venous return and cardiac filling without postural change (Goswami et al., 2019). When applied in the supine position, LBNP reduces SV while mean arterial pressure (MAP) is often maintained within the autoregulatory range via reflex adjustments in HR and peripheral vascular resistance. Standard LBNP paradigms typically employ an off‐feet posture to minimize activation of the skeletal muscle pump, such that the resulting responses are driven predominantly by isometric contraction and arterial baroreflex unloading (Goswami et al., 2019). Compared with upright head‐up tilt, supine LBNP avoids changes in the heart–brain hydrostatic gradient and maintains head orientation, thereby minimizing confounding effects of vestibular (otolithic) input on autonomic outflow (Ray, 2000).

The skeletal muscle pump is recognized as an important modulator of venous return and cardiac filling during orthostatic and hypovolaemic challenges. Previous studies have shown that lower‐limb muscle activation or external compression can influence central haemodynamic responses during LBNP and orthostatic tolerance (Hockin & Claydon, 2019; Hockin et al., 2019; Watenpaugh et al., 1994). These findings establish that engaging the lower limbs can modify systemic cardiovascular responses during central hypovolaemia. However, in these paradigms, the implications for CBV regulation under conditions in which posture, the heart–brain hydrostatic gradient and vestibular input are held constant remain less well‐defined.

Accordingly, the present study employed an on‐feet LBNP paradigm in which participants, while supine, engaged in static lower‐limb isometric bracing against a footplate. This configuration allowed lower‐limb muscle engagement to be manipulated without altering posture, the hydrostatic column, or otolith‐mediated vestibular input. By comparing on‐feet and off‐feet conditions across graded levels of LBNP in a randomized, within‐subject design, we tested the hypothesis that isometric lower‐limb engagement during supine LBNP would be associated with preservation of CBV, consistent with preload‐related effects inferred from changes in SV, while alterations in HR and systemic vascular conductance (SVC) reflect secondary compensatory responses rather than independent determinants of CBV.

## 2 METHODS

### 2.1 Subjects

Fourteen healthy adults (age: 25 ± 5 years; 5 women; height: 165.2 ± 8.0 cm; weight: 60.6 ± 10.6 kg) voluntarily participated in this study. All participants were non‐smokers, free of any known cardiovascular or cerebrovascular disease, and not taking any medications known to affect systemic or cerebrovascular function. Prior to participation, written informed consent was obtained from each subject after a detailed explanation of all potential risks. One male participant taller than 185 cm was excluded due to exceeding the size limit of the LBNP chamber. Consequently, 13 subjects were included in the final analysis. A priori power analysis based on our previous study (Ninomiya et al., 2019) indicated that a sample size of 12 participants per condition would provide 95% power to detect changes in SV and mean CBV at the middle cerebral artery velocity with an effect size of 1.15 and 0.501, and an α level of <0.05. All experimental procedures conformed to the principles outlined in the *Declaration of Helsinki* and were approved by the institutional research ethics board at the National Institute of Advanced Industrial Science and Technology (AIST) (approval No. 2018–862).

### 2.2 Experimental protocol

Participants fasted and abstained from caffeinated beverages for ≥3 h and from alcohol and strenuous physical activity for 24 h before testing. Upon arrival, height and weight were measured. After ≥20 min of supine rest inside the LBNP chamber, two conditions were performed in randomized order: the off‐feet (e.g., relaxed legs) condition and the on‐feet (bracing feet) condition. In the off‐feet condition, the participant was positioned on a saddle to prevent caudal displacement. Surface electromyography (EMG) of the lower‐limb muscles was recorded during the protocol. However, EMG signals were not quantitatively analysed to confirm complete muscle inactivity during the off‐feet condition. Participants were instructed to keep their lower‐limb muscles relaxed, and compliance was monitored by continuous visual inspection throughout the experiment. In the on‐feet condition, the participant resisted the caudal force by pressing both feet against a rigid footplate with slight knee flexion (isometric bracing). A minimum interval of 5 min was allowed between experimental conditions. The second condition was initiated only after systemic haemodynamic variables had returned to baseline levels. In each condition, LBNP was applied in fixed stages of −30 mmHg followed by −50 mmHg, each for 6 min. Termination criteria for LBNP exposure included: (1) systolic blood pressure <80 mmHg; (2) systolic blood pressure <90 mmHg accompanied by presyncopal symptoms (e.g., lightheadedness, nausea, sweating); or (3) participant request due to presyncopal symptoms (Hirabuki et al., 2025). No participant reached these termination criteria during the −50 mmHg LBNP stage. One participant declined to perform the −50 mmHg trial in the off‐feet condition due to fear of syncope and withdrew from that stage.

### 2.3 LBNP setup

A custom cylindrical LBNP chamber (inner diameter 40 cm; polyvinyl chloride) was used. Participants lay supine without head or neck support; no pillow was used during the protocol. Their abdomen was sealed at the level of the iliac crests using a neoprene waist seal. Negative pressure was generated by a vacuum pump and manually regulated via a valve while monitoring a pressure transducer mounted on the chamber wall at ankle level; pressure was referenced to ambient and continuously recorded. Before each trial, the system was leak‐checked; step changes to −30 and −50 mmHg were achieved within ∼5 s. The chamber's inner wall provided a low‐friction interface to minimize shear. Pilot measurements, informed by the chamber inner diameter and the stretch properties of the neoprene waist seal, confirmed that the load transmitted to the footplate averaged ∼28 kgf at −30 mmHg and ∼70 kgf at −50 mmHg.

### 2.4 Systemic and cerebral haemodynamic measurements

HR was obtained from electrocardiographic recordings using a three‐lead system (ML132 Bio Amp, ADInstruments, Colorado Springs, CO, USA). Radial arterial pressure was assessed using applanation tonometry (Jentow, Nihon Colin, Komaki, Japan) because the protocol was part of a broader experimental series requiring estimation of central (aortic) pressure. Radial arterial pressure waveforms were recorded at the right wrist and calibrated against brachial cuff blood pressure measured by oscillometry. SV was estimated from the radial arterial pressure waveforms using the Modelflow method (BeatScope 1.1a, Finapres Medical Systems, Amsterdam, the Netherlands) (Sugawara et al., 2003; Wesseling et al., 1993), and CO was calculated as the product of SV and HR. SVC was derived by dividing CO by MAP. CBV was continuously measured in the right middle cerebral artery (MCA) through the ipsilateral temporal window using a 2‐MHz transcranial Doppler probe (EzDop; DWL, Sipplingen, Germany). The transcranial Doppler probe was fixed in place using a commercially available headband, and insonation depth and signal quality were confirmed prior to data collection and maintained throughout the protocol. The sample depth (42–55 mm) and probe angle were adjusted individually at the start of the experiment to optimize signal quality according to standard procedures (Aaslid et al., 1982). The time‐average mean and peak and minimum CBV were obtained. Cerebral vascular conductance index (CVCi) was calculated by dividing mean CBV by MAP. End‐tidal CO_2_ ($ET_{CO_{2}}$) was monitored with a capnometer (OMG‐3800, Nihon Koden, Tokyo, Japan). Respiratory rate was not directly assessed during the protocol. Subjects were instructed to breathe normally during data collection.

### 2.5 Statistics

Averaged values from the final 30 s of each stage were analysed. Linear mixed models (LMM) were used to assess the main effects of LBNP (baseline, −30 mmHg and −50 mmHg) and leg muscle condition (off‐feet and on‐feet), as well as their interaction. LBNP‐induced changes (Δ from baseline) were also analysed with LMMs using the same fixed‐ and random‐effects structure. Models included random intercepts and random slopes for LBNP intensity by participant, with a compound‐symmetry covariance structure to account for within‐subject correlations across pressure levels. Parameter estimation used maximum likelihood. When significant main or interaction effects were detected, Bonferroni‐adjusted *post hoc* comparisons were conducted. Intraindividual associations between variables of interest were examined using repeated‐measures correlation analysis (rmcorr) in R (Bakdash & Marusich, 2017). Additionally, LMM was used to examine associations between SV and CBV, with participant included as a random intercept to account for within‐subject repeated measures and end‐tidal CO_2_ included as a covariate. Data are reported as estimated marginal means with 95% confidence intervals (CI), and statistical significance was set a priori at *P* < 0.05.

## 3 RESULTS

LMM analyses were conducted on data from 13 participants; one female participant did not complete the −50 mmHg LBNP stage in the off‐feet condition, not due to presyncope, but because she declined to proceed owing to concern about potential syncope.

**FIGURE 1:**
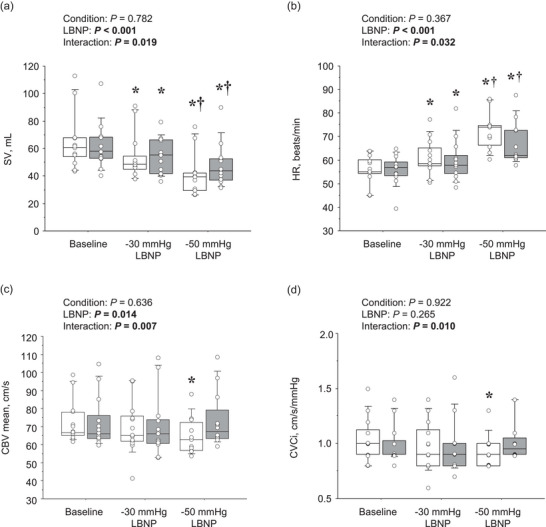
Responses of systemic and cerebral haemodynamics to lower‐body negative pressure (LBNP). (a) Stroke volume (SV); (b) heart rate (HR); (c) mean middle cerebral artery blood velocity (CBV mean); (d) cerebrovascular conductance index (CVCi). Boxes indicate medians and interquartile ranges; whiskers depict data spread; individual points represent participant values. *Significant difference from the baseline; **†**significant difference from −30 mmHg.

**FIGURE 2:**
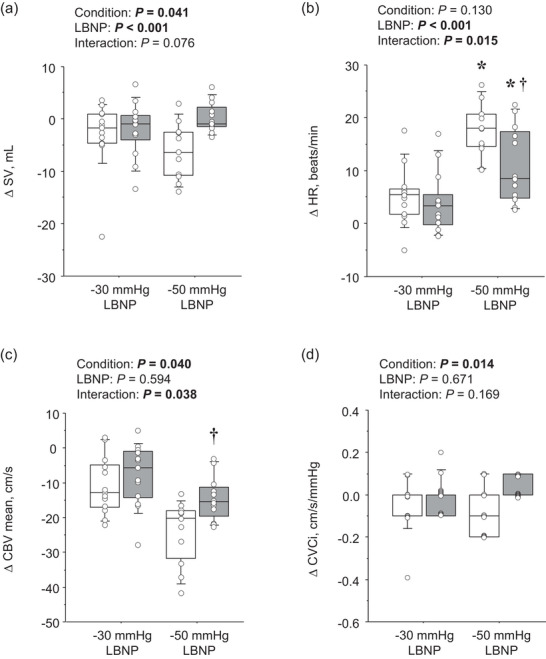
Comparisons of LBNP‐induced changes (Δ from baseline) in systemic and cerebral haemodynamics between off‐ and on‐feet conditions. (a) Stroke volume (SV); (b) heart rate (HR); (c) mean middle cerebral artery blood velocity (CBV mean); (d) cerebrovascular conductance index (CVCi). Boxes indicate medians and interquartile ranges; whiskers depict data spread; individual points represent participant values. *Significant difference from −30 mmHg; **†**significant difference from the off‐feet condition.

Table 1 summarizes systemic and cerebral haemodynamic responses to LBNP. Systolic BP decreased progressively with increasing LBNP intensity (*P* < 0.001), whereas diastolic BP and MAP remained unchanged (both *P* > 0.05). No significant main effect of feet condition or LBNP × feet interaction was observed. SV declined significantly with LBNP in both conditions (*P* < 0.001, Figure 1a), but the reduction was consistently smaller in the on‐feet than in the off‐feet condition (main effect of feet: *P* = 0.041; interaction: *P* = 0.076; Figure 2a). Conversely, HR increased with LBNP (main effect of LBNP: *P* < 0.0001), and this rise was attenuated in the on‐feet condition (interaction: *P* = 0.032; Figure 1b). *Post hoc* testing confirmed that at −50 mmHg, the increase in HR was significantly smaller in the on‐feet than in the off‐feet condition (*P* = 0.022, Figure 2b). CO and SVC decreased similarly across conditions (main effect of LBNP: *P* ≤ 0.001 for both) with no significant condition or interaction effects. When comparing LBNP‐induced changes (Δ from baseline), CO showed a trend toward a larger reduction at −50 than at −30 mmHg (*P* = 0.062), with no significant foot‐condition effect (*P* = 0.161). By contrast, SVC tended to be reduced more off‐feet than on‐feet (*P* = 0.064), with no significant difference between −30 and −50 mmHg (*P* = 0.268).

**TABLE 1:** Systemic and cerebral haemodynamic responses to lower body negative pressure stimulation.

		EMM 95% CI (lower, upper)		LMM		
	LBNP	Off‐feet	On‐feet	Effect	*P*	Partial η^2^
MAP (mmHg)	0 mmHg	72 (69, 74)	74 (71, 77)	C	0.360	0.035
	−30 mmHg	72 (69, 75)	73 (70, 76)	L	0.067	0.112
	−50 mmHg	70 (67, 73)	72 (69, 75)	C×L	0.489	0.031
SBP (mmHg)	0 mmHg	104 (100, 109)	107 (103, 111)	C	0.413	0.028
	−30 mmHg	104 (100, 109)	105 (100, 109)	L	**<0.001**	0.333
	−50 mmHg	97 (93, 102)	101 (96, 105)	C×L	0.571	0.024
DBP (mmHg)	0 mmHg	57 (54, 60)	60 (57, 64)	C	0.458	0.023
	−30 mmHg	59 (56, 62)	60 (57, 63)	L	0.485	0.031
	−50 mmHg	60 (57, 63)	60 (57, 63)	C×L	0.171	0.075
CO (L/min)	0 mmHg	3.6 (3.0, 4.1)	3.5 (2.9, 4.0)	C	0.920	0.000
	−30 mmHg	3.3 (2.7, 3.8)	3.3 (2.7, 3.8)	L	**<0.001**	0.357
	−50 mmHg	2.9 (2.4, 3.5)	3.1 (2.6, 3.7)	C×L	0.293	0.052
SVC (ml/min/mmHg)	0 mmHg	50.2 (42.1, 58.4)	47.0 (38.9, 55.2)	C	0.934	0.000
	−30 mmHg	45.0 (36.9, 53.2)	45.0 (36.8, 53.1)	L	**0.001**	0.259
	−50 mmHg	42.3 (34.0, 50.5)	44.2 (36.0, 52.4)	C×L	0.180	0.072
CBV_peak_ (cm/s)	0 mmHg	106 (96, 116)	107 (96, 117)	C	0.558	0.014
	−30 mmHg	100 (90, 110)*	102 (92, 112)	L	**<0.001**	0.431
	−50 mmHg	92 (82, 103)*^†^	102 (92, 113)	C*L	**0.008**	0.183
CBV_min_ (cm/s)	0 mmHg	52 (46, 58)	52 (46, 57)	C	0.805	0.002
	−30 mmHg	52 (46, 58)	52 (46, 58)	L	0.078	0.108
	−50 mmHg	53 (47, 58)	56 (50, 61)	C*L	0.265	0.058
$ET_{CO_{2}}$ (%)	0 mmHg	38.0 (36.7, 39.4)	37.6 (36.3, 38.9)	C	0.729	0.005
	−30 mmHg	36.6 (35.3, 37.9)	36.7 (35.3, 38.0)	L	**<0.001**	0.394
	−50 mmHg	36.1 (34.7, 37.4)	35.5 (34.2, 36.9)	C*L	0.675	0.017

Data are estimated marginal means and 95% confidence intervals (CI). C, main effect of foot condition (off‐feet, on‐feet); L, main effect of LBNP (baseline, −30 mmHg, −50 mmHg); C×L, interaction between foot conditions and LBNP. *P*‐values shown in bold indicate statistical significance. *P < 0.05 vs. baseline of the same foot condition. ^†^ *P* < 0.05 vs. −30 mmHg of the same foot condition. CBV_min_, minimal cerebral blood velocity; CBV_peak_, peak cerebral blood velocity; CO, cardiac output; DBP, diastolic blood pressure; EMM, estimated marginal means; $ET_{CO_{2}}$, end‐tidal carbon dioxide; LBNP, lower body negative pressure; LMM, linear mixed model; MAP, mean arterial pressure; SBP, systolic blood pressure; SVC, systemic vascular conductance.

Peak and mean CBV decreased significantly with LBNP in the off‐feet condition, whereas they were preserved across LBNP intensities in the on‐feet condition (interaction: *P* = 0.008 and *P* = 0.007, respectively; Table 1 and Figure 1c). Consistent with this, CVCi declined substantially in the off‐feet condition but remained preserved in the on‐feet condition (interaction: *P* = 0.010; Figure 1d). When comparing LBNP‐induced changes, mean CBV showed a smaller on‐feet reduction at −50 mmHg (interaction *P* = 0.038; Bonferroni *P* = 0.004; Figure 2c). CVCi was likewise attenuated on‐feet (*P* = 0.014); at −50 mmHg it rose slightly on‐feet and fell off‐feet, with no significant LBNP × foot interaction (*P* = 0.169; Figure 2d). Although $ET_{CO_{2}}$ decreased significantly with LBNP (*P* < 0.001, Table 1), this response was not influenced by feet condition (main effect of feet: *P* = 0.729; interaction: *P* = 0.675).

**FIGURE 3:**
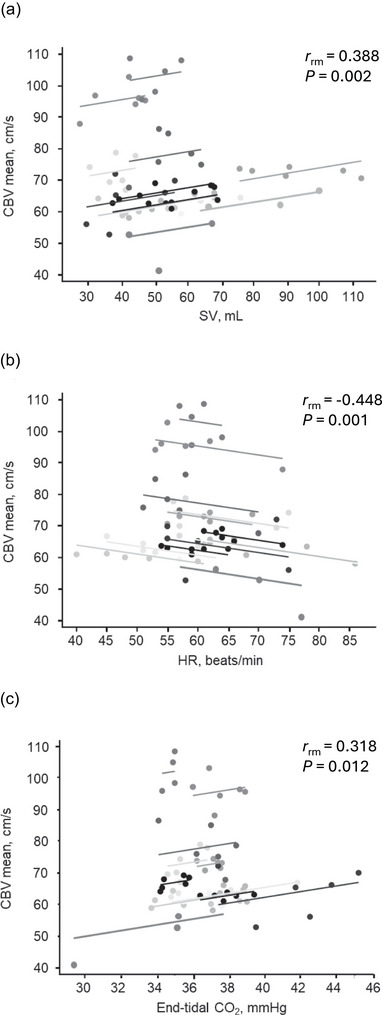
Repeated‐measures correlation (rmcorr) between mean middle cerebral artery blood velocity (CBV mean) and (a) stroke volume (SV), (b) heart rate (HR), and (c) end‐tidal CO_2_ ($ET_{CO_{2}}$). Points are repeated observations greyscaled by participant. Lines are participant‐specific fits constrained to a common slope estimated by rmcorr (i.e., parallel lines with participant‐specific intercepts).

Repeated‐measures correlation analyses showed that mean CBV was significantly associated with SV (*r*_rm_ = 0.388, *P* = 0.002), HR (*r*_rm_ = −0.448, *P* = 0.001) and $ET_{CO_{2}}$ (*r*_rm_ = 0.318, *P* = 0.012) (Figure 3). In contrast, no significant correlations were observed with MAP (*r*_rm_ = −0.003, *P* = 0.980), CO (*r*_rm_ = 0.237, *P* = 0.062) and SVC (*r*_rm_ = 0.193, *P* = 0.129). To account for the influence of $ET_{CO_{2}}$, we fitted the LMM with a participant included as a random intercept. After adjustment for $ET_{CO_{2}}$, SV remained significantly associated with mean CBV (*P* = 0.011), whereas $ET_{CO_{2}}$ was not a significant predictor of CBV (*P* = 0.19).

## 4 DISCUSSION

The main findings of this study are that CBV and CVCi were maintained across LBNP intensities in the on‐feet condition, in contrast to their marked reductions in the off‐feet condition. Supporting this, SV declined less and the compensatory rise in HR was blunted in the on‐feet condition. Importantly, MAP was maintained in both conditions across all LBNP stages, indicating that the observed CBV responses occurred without a fall in perfusion pressure. Consistent with this, repeated‐measures correlations showed that CBV tracked changes in SV, HR and end‐tidal CO_2_, but not MAP. Together, these findings are consistent with the interpretation that, within a maintained‐MAP, autoregulatory range, preload preservation contributes to the maintenance of CBV during on‐feet bracing under LBNP, while increases in HR likely reflect compensatory responses to changes in SV rather than primary drivers of CBV.

Conceptually, cerebral blood flow regulation reflects the combined action of cerebrovascular autoregulation and central circulatory drive (Claassen et al., 2021; Schondorf et al., 2001; Stewart, 2012). In particular, CO exerts a measurable influence on cerebral blood flow (Ogoh et al., 2005; Willie et al., 2014), indicating that cerebral blood flow is not solely determined by arterial pressure within the autoregulatory range, but is also modulated by other factors such as arterial CO_2_ tension and intracranial pressure. Conceptually, central/systemic haemodynamics are governed by two primary components: a cardiac component (SV, HR) and a vascular component (resistance/conductance) (Rowell, 1993). Accordingly, we hypothesized that preserving preload, as inferred from changes in SV, would contribute to the maintenance of CBV during LBNP.

During orthostatic stress, gravitational pooling reduces venous return and preload, leading to a fall in SV (Cooke et al., 2004; Rowell, 1993). This reduction in cardiac filling is sensed by arterial baroreceptors, eliciting a compensatory increase in HR and peripheral vascular resistance to defend CO and arterial pressure (Cooke et al., 2004; Rowell, 1993). In the off‐feet condition, a compensatory rise in HR was observed in response to the reduction in SV; however, this adjustment was insufficient to maintain CO, resulting in a reduction in CBV (Rickards et al., 2015). Although CO was reduced in both conditions, CBV declined only in the off‐feet condition, likely reflecting a greater reduction in preload and cerebral vascular conductance. In contrast, on‐feet bracing attenuated preload loss and preserved cerebral vascular conductance, allowing CBV to be maintained despite reduced systemic flow within a maintained autoregulatory range.

Within this framework, in the on‐feet condition, the LBNP‐induced reduction in SV was attenuated. By engaging the leg muscles against the footplate, participants generated a muscle‐pump effect, even in a static manner, that counteracted venous pooling in the lower body. Although this lower‐limb engagement was static rather than rhythmic, the resulting preload‐supporting effect should be distinguished from a classical cyclical skeletal muscle pump and instead reflects sustained isometric muscle tension. This sustained atrial filling mitigated cardiopulmonary baroreceptor unloading and blunted the reflexive rise in HR.

The attenuated increase in HR in the on‐feet condition can be regarded as physiologically advantageous. While tachycardia is a compensatory response to reduced SV, pronounced increases shorten diastolic filling time and may impair coronary and cerebral perfusion (Heusch, 2008). With better SV preservation via the muscle pump, the need for marked HR elevation was reduced, limiting cardiac strain – a feature consistent with a more efficient circulatory adjustment.

Turning to the vascular component, there was a tendency for a smaller reduction in SVC in the off‐feet than the on‐feet condition, consistent with stronger sympathetic vasoconstrictor engagement when preload was compromised (Fu et al., 2006). However, changes in CBV were not significantly associated with changes in SVC, suggesting that augmented peripheral vasoconstriction alone does not account for the observed differences in CBV between conditions.

Notably, MAP was maintained in both conditions across LBNP stages. In keeping with this haemodynamic stability, repeated‐measures correlations showed that CBV covaried with SV and $ET_{CO_{2}}$, but not with MAP – a pattern compatible with effective autoregulatory buffering of modest pressure fluctuations. Although these associations were modest in magnitude, they are consistent with the interpretation that preload‐related effects, rather than arterial pressure per se or augmented peripheral vasoconstriction, contribute to the maintenance of CBV during central hypovolaemia. This interpretation accords with Levine et al. (1991), who demonstrated that endurance athletes, owing to enhanced ventricular compliance, operate on the steep portion of the Starling curve and therefore exhibit exaggerated reductions in SV during central hypovolaemia. Consistent with prior work demonstrating that lower‐limb muscle activity or compression can influence cardiovascular responses during LBNP (Hockin & Claydon, 2019; Hockin et al., 2019; Watenpaugh et al., 1994), the present findings extend this literature by evaluating CBV responses in a supine paradigm that minimizes confounding effects of posture and vestibular activation.

### 4.1 Clinical and translational implications

Results of this study align with prior evidence that simple isometric counterpressure manoeuvres (e.g. leg crossing with muscle tensing) raise arterial pressure and improve orthostatic tolerance, with guideline support in prodromal vasovagal syncope and practical applicability in older adults with orthostatic hypotension (Brignole et al., 2018; Dani et al., 2021; Freeman et al., 2018). Within this context, our results are consistent with the notion that preload preservation, rather than augmented peripheral vasoconstriction, contributes to the maintenance of CBV during central hypovolaemia. Such strategies may be particularly relevant for older adults, who commonly exhibit reduced venous return and impaired autonomic reflexes, as well as for patients with recurrent vasovagal syncope or orthostatic hypotension. Beyond these clinical populations, active lower‐limb muscle engagement may also serve as a simple and practical countermeasure against post‐flight orthostatic intolerance in astronauts, in whom vascular deconditioning and central blood volume redistribution are prominent contributors.

### 4.2 Methodological strengths

It should be acknowledged that head‐up tilt has the major advantage of reflecting actual orthostasis and therefore may have greater clinical relevance for patients with orthostatic intolerance. The present supine LBNP approach was not intended to replace upright paradigms, but rather to provide a physiologically controlled framework in which the effects of lower‐limb muscle engagement on preload and cerebral blood velocity could be examined without concurrent changes in posture or hydrostatic loading.

It should also be noted that many head‐up tilt protocols include a footboard, which may permit some degree of static lower‐limb muscle activation. However, in upright tilt, this muscle engagement occurs concurrently with postural change, hydrostatic redistribution and vestibular activation, making it difficult to isolate its specific contribution. In contrast, the on‐feet LBNP paradigm allowed lower‐limb muscle engagement to be manipulated independently of these factors.

Passive head‐up tilt (HUT) alters the heart–brain hydrostatic gradient, thereby changing cerebral perfusion pressure (CPP), which complicates isolation of central haemodynamic mechanisms (Bronzwaer et al., 2017; Claassen et al., 2021). By contrast, the supine LBNP protocol maintained a constant heart–brain hydrostatic gradient – and thus CPP – while selectively introducing lower‐limb muscle engagement via footplate loading (on‐feet isometric bracing). Whereas standard passive HUT largely suppresses the skeletal muscle pump, the on‐feet LBNP configuration allowed controlled modulation of lower‐limb activation without altering the hydrostatic column.

A further limitation of HUT is the concomitant activation of otolith afferents, which can additively augment muscle sympathetic nerve activity; supine LBNP maintains head position and leaves otolith input essentially unchanged, isolating baroreflex‐ and preload‐mediated responses (Ray, 2000). Additional strengths include the randomized, within‐subject comparison of on‐feet and off‐feet conditions, which effectively toggled lower‐limb muscle engagement while holding posture constant. With MAP maintained across LBNP stages, repeated‐measures analyses indicated that changes in CBV were more closely associated with SV and $ET_{CO_{2}}$ than with arterial pressure. Collectively, this design provides a physiologically controlled framework for examining the relative contribution of preload preservation, as distinct from augmented peripheral vasoconstriction, to CBV regulation during central hypovolaemia.

### 4.3 Limitations and future directions

Despite the high degree of experimental control afforded by the on‐feet LBNP paradigm, several limitations should be acknowledged. First, we did not obtain direct measures of global cerebral inflow (e.g., internal carotid or vertebral artery flow), nor did we assess central venous pressure or thoracic blood volume. Consequently, the present findings are based on changes in CBV and systemic haemodynamics and do not permit definitive conclusions regarding absolute cerebral blood flow or preload per se. Second, CBV was assessed using transcranial Doppler ultrasound of the middle cerebral artery. Although this approach is widely used in LBNP research, it assumes a stable arterial diameter and does not capture volumetric cerebral blood flow. Accordingly, inferences regarding cerebral perfusion should be interpreted cautiously. Future studies incorporating duplex ultrasound or imaging‐based assessments of cerebral inflow would help clarify whether preload‐preserving strategies maintain global cerebral blood flow under central hypovolaemia. Third, although end‐tidal CO_2_ was continuously monitored, it was not experimentally clamped. Respiratory rate was not measured, and therefore subtle changes in breathing pattern that may have influenced end‐tidal CO_2_ cannot be excluded. Given the potent influence of arterial CO_2_ on cerebrovascular tone, such fluctuations may have contributed to variability in CBV responses. While repeated‐measures analyses accounted for $ET_{CO_{2}}$, statistical adjustment cannot fully substitute for controlled manipulation. Fourth, SV was estimated using a non‐invasive Modelflow approach. Although this method provides reliable indices of relative change (Sugawara et al., 2003), SV reflects the combined influence of preload, afterload and cardiac contractility, and does not directly quantify preload. Thus, interpretations linking SV changes to preload should be considered indirect. In addition, because cardiac output was derived from Modelflow‐based SV estimates, the absolute CO values should likewise be interpreted cautiously, with emphasis placed on relative within‐subject and between‐condition differences rather than absolute magnitude. Fifth, participants were instructed to abstain from caffeine for at least 3 h before testing. Although this duration is commonly used in experimental studies, residual effects of caffeine cannot be entirely excluded and may have contributed to interindividual variability in cardiovascular or cerebrovascular responses. This potential influence should be considered when interpreting the present findings. Sixth, although lower‐limb EMG was recorded, we did not quantitatively verify the absence of muscle activity during the off‐feet condition, and subtle tonic activation cannot be entirely excluded. Finally, the sample comprised a modest number of healthy young adults, and menstrual cycle phase or hormonal status was not controlled among female participants. These factors may influence autonomic and cerebrovascular regulation and limit generalizability. Future work should extend this approach to older adults and patient populations with orthostatic disorders and incorporate direct assessments of central blood volume and cerebral inflow to further refine mechanistic understanding. Supporting Information

### 4.4 Conclusion

In conclusion, this study demonstrates that on‐feet bracing is associated with preservation of CBV during LBNP, consistent with a preload‐mediated effect rather than reliance on augmented peripheral vasoconstriction. The accompanying HR responses appear to reflect compensatory adjustments rather than primary drivers of CBV within a maintained‐MAP, autoregulatory range. While primarily physiological, these findings may also inform future strategies to mitigate orthostatic intolerance in susceptible populations.

## AUTHOR CONTRIBUTIONS

Jun Sugawara designed the study. Jun Sugawara, Marina Fukuie and Tsubasa Tomoto performed experimentsand analysed the data. All authors interpreted the results of the experiments. Jun Sugawara wrote the draft of the manuscript. All authors edited and revised the final version of the manuscript. All authors have read and approved the final version of this manuscript and agree to be accountable for all aspects of the work in ensuring that questions related to the accuracy or integrity of any part of the work are appropriately investigated and resolved. All persons designated as authors qualify for authorship, and all those who qualify for authorship are listed.

## CONFLICT OF INTEREST

The authors declare no conflicts of interest.

## Supporting information



Supporting Tables 1 and 2.

## Data Availability

Data will be made available upon request to the lead contact.
