# Supplementary material for: On‐feet isometric bracing maintains cerebral arterial blood velocity during lower body negative pressure via preload augmentation
Source: Exp Physiol. 2026 Feb 4;111(4):1756–65. doi: 10.1113/EP093648 (PMC13140660; doi:10.1113/EP093648)
Supplement: Supplementary file 1 — Supporting Tables 1 and 2. [file EPH-111-1756-s001.docx]

Supplemental Table 1. Hemodynamic responses to lower body negative pressure (LBNP) stimulation.

|  |  | Off-feet | | | | |  | On-feet | | | | |  | LMM | | |
| --- | --- | --- | --- | --- | --- | --- | --- | --- | --- | --- | --- | --- | --- | --- | --- | --- |
|  |  |  |  | 95% CI | |  |  |  |  | 95% CI | |  |  |  |  | Partial |
|  | LBNP | EMM | ( | lower | upper | ) |  | EMM | ( | lower | upper | ) |  | Effect | P-value | η^2^ |
| MAP | 0 mmHg | 72 | ( | 69 | 74 | ) |  | 74 | ( | 71 | 77 | ) |  | C | 0.360 | 0.035 |
| mmHg | -30mmHg | 72 | ( | 69 | 75 | ) |  | 73 | ( | 70 | 76 | ) |  | L | 0.067 | 0.112 |
|  | -50mmHg | 70 | ( | 67 | 73 | ) |  | 72 | ( | 69 | 75 | ) |  | C*L | 0.489 | 0.031 |
| SBP | 0 mmHg | 104 | ( | 100 | 109 | ) |  | 107 | ( | 103 | 111 | ) |  | C | 0.413 | 0.028 |
| mmHg | -30mmHg | 104 | ( | 100 | 109 | ) |  | 105 | ( | 100 | 109 | ) |  | L | **< 0.001** | 0.333 |
|  | -50mmHg | 97 | ( | 93 | 102 | ) |  | 101 | ( | 96 | 105 | ) |  | C*L | 0.571 | 0.024 |
| DBP | 0 mmHg | 57 | ( | 54 | 60 | ) |  | 60 | ( | 57 | 64 | ) |  | C | 0.458 | 0.023 |
| mmHg | -30mmHg | 59 | ( | 56 | 62 | ) |  | 60 | ( | 57 | 63 | ) |  | L | 0.485 | 0.031 |
|  | -50mmHg | 60 | ( | 57 | 63 | ) |  | 60 | ( | 57 | 63 | ) |  | C*L | 0.171 | 0.075 |
| HR | 0 mmHg | 56 | ( | 52 | 60 | ) |  | 56 | ( | 52 | 60 | ) |  | C | 0.367 | 0.034 |
| beat/min | -30mmHg | 61 | ( | 57 | 65 | ) |  | 60 | ( | 56 | 64 | ) |  | L | **< 0.001** | 0.777 |
|  | -50mmHg | 73 | ( | 69 | 78 | ) |  | 67 | ( | 63 | 72 | ) |  | C*L | **0.032** | 0.142 |
| SV | 0 mmHg | 65 | ( | 55 | 74 | ) |  | 62 | ( | 53 | 71 | ) |  | C | 0.782 | 0.003 |
| ml | -30mmHg | 54 | ( | 45 | 63 | ) |  | 54 | ( | 45 | 63 | ) |  | L | **< 0.001** | 0.728 |
|  | -50mmHg | 40 | ( | 31 | 50 | ) |  | 47 | ( | 38 | 57 | ) |  | C*L | **0.019** | 0.135 |
| CO | 0 mmHg | 3.6 | ( | 3.0 | 4.1 | ) |  | 3.5 | ( | 2.9 | 4.0 | ) |  | C | 0.920 | 0.000 |
| L/min | -30mmHg | 3.3 | ( | 2.7 | 3.8 | ) |  | 3.3 | ( | 2.7 | 3.8 | ) |  | L | **< 0.001** | 0.357 |
|  | -50mmHg | 2.9 | ( | 2.4 | 3.5 | ) |  | 3.1 | ( | 2.6 | 3.7 | ) |  | C*L | 0.293 | 0.052 |
| SVC | 0 mmHg | 50.2 | ( | 42.1 | 58.4 | ) |  | 47.0 | ( | 38.9 | 55.2 | ) |  | C | 0.934 | 0.000 |
| ml/min/mmHg | -30mmHg | 45.0 | ( | 36.9 | 53.2 | ) |  | 45.0 | ( | 36.8 | 53.1 | ) |  | L | **0.001** | 0.259 |
|  | -50mmHg | 42.3 | ( | 34.0 | 50.5 | ) |  | 44.2 | ( | 36.0 | 52.4 | ) |  | C*L | 0.180 | 0.072 |
| CBV mean | 0 mmHg | 72 | ( | 65 | 80 | ) |  | 72 | ( | 65 | 80 | ) |  | C | 0.636 | 0.007 |
| cm/sec | -30mmHg | 70 | ( | 62 | 77 | ) |  | 70 | ( | 63 | 78 | ) |  | L | **0.014** | 0.180 |
|  | -50mmHg | 65 | ( | 58 | 73 | ) |  | 72 | ( | 65 | 80 | ) |  | C*L | **0.007** | 0.208 |
| CBV peak | 0 mmHg | 106 | ( | 96 | 116 | ) |  | 107 | ( | 96 | 117 | ) |  | C | 0.558 | 0.014 |
| cm/sec | -30mmHg | 100 | ( | 90 | 110 | ) |  | 102 | ( | 92 | 112 | ) |  | L | **< 0.001** | 0.431 |
|  | -50mmHg | 92 | ( | 82 | 103 | ) |  | 102 | ( | 92 | 113 | ) |  | C*L | **0.008** | 0.183 |
| CBV min | 0 mmHg | 52 | ( | 46 | 58 | ) |  | 52 | ( | 46 | 57 | ) |  | C | 0.805 | 0.002 |
| cm/sec | -30mmHg | 52 | ( | 46 | 58 | ) |  | 52 | ( | 46 | 58 | ) |  | L | 0.078 | 0.108 |
|  | -50mmHg | 53 | ( | 47 | 58 | ) |  | 56 | ( | 50 | 61 | ) |  | C*L | 0.265 | 0.058 |
| CVCi | 0 mmHg | 1.02 | ( | 0.91 | 1.14 | ) |  | 0.97 | ( | 0.86 | 1.09 | ) |  | C | 0.922 | 0.000 |
| cm/sec/mmHg | -30mmHg | 0.97 | ( | 0.85 | 1.09 | ) |  | 0.96 | ( | 0.85 | 1.08 | ) |  | L | 0.265 | 0.049 |
|  | -50mmHg | 0.93 | ( | 0.81 | 1.05 | ) |  | 1.01 | ( | 0.89 | 1.13 | ) |  | C*L | **0.010** | 0.161 |
| ETCO_2_ | 0 mmHg | 38.0 | ( | 36.7 | 39.4 | ) |  | 37.6 | ( | 36.3 | 38.9 | ) |  | C | 0.729 | 0.005 |
| % | -30mmHg | 36.6 | ( | 35.3 | 37.9 | ) |  | 36.7 | ( | 35.3 | 38.0 | ) |  | L | **< 0.001** | 0.394 |
|  | -50mmHg | 36.1 | ( | 34.7 | 37.4 | ) |  | 35.5 | ( | 34.2 | 36.9 | ) |  | C*L | 0.675 | 0.017 |

Data are estimated marginal means (EMM) and 95% confidence intervals (CI). “C”, main effect of foot condition (off-feet, on-feet); “L”, main effect of LBNP (baseline, -30 mmHg, -50 mmHg); “C*L”, interaction between foot conditions and LBNP; MAP, mean arterial pressure; SBP, systolic blood pressure; DBP, diastolic blood pressure; CO, cardiac output; SVC, systemic vascular conductance; CBV peak, peak cerebral blood velocity (CBV); CBV min, minimal CBV; EtCO_2_, end-tidal carbon dioxide. *: P < 0.05 vs. baseline of the same foot condition. ^†^: P < 0.05 vs. -30 mmHg of the same foot condition.

Supplemental Table 2. Lower body negative pressure (LBNP)-induced changes in systemic and cerebral hemodynamics.

|  |  | Off-feet | | | | |  | On-feet | | | | |  | LMM | | |
| --- | --- | --- | --- | --- | --- | --- | --- | --- | --- | --- | --- | --- | --- | --- | --- | --- |
|  |  |  | 95% CI | | | |  |  | 95% CI | | | |  |  |  | Partial |
|  |  | EMM | ( | lower, upper | | ) |  | EMM | ( | lower, upper | | ) |  | Effect | P-value | η^2^ |
| ΔMAP | -30mmHg | 0.9 | ( | -1.5 | 3.2 | ) |  | -1.3 | ( | -3.6 | 1.1 | ) |  | C | 0.185 | 0.073 |
| mmHg | -50mmHg | -1.6 | ( | -4.1 | 1.0 | ) |  | -3.0 | ( | -5.4 | -0.5 | ) |  | L | 0.077 | 0.129 |
|  |  |  |  |  |  | ) |  |  |  |  |  |  |  | C*L | 0.736 | 0.005 |
| ΔSBP | -30mmHg | -0.2 | ( | -3.9 | 3.6 | ) |  | -2.4 | ( | -6.1 | 1.3 | ) |  | C | 0.718 | 0.005 |
| mmHg | -50mmHg | -6.9 | ( | -10.9 | -2.9 | ) |  | -6.2 | ( | -10.1 | -2.4 | ) |  | L | **0.003** | 0.320 |
|  |  |  |  |  |  |  |  |  |  |  |  |  |  | C*L | 0.378 | 0.034 |
| ΔDBP | -30mmHg | 2.0 | ( | -0.2 | 4.2 | ) |  | -0.3 | ( | -2.5 | 1.9 | ) |  | C | **0.027** | 0.190 |
| mmHg | -50mmHg | 2.4 | ( | 0.0 | 4.8 | ) |  | -0.8 | ( | -3.0 | 1.5 | ) |  | L | 0.976 | 0.000 |
|  |  |  |  |  |  |  |  |  |  |  |  |  |  | C*L | 0.687 | 0.007 |
| ΔHR | -30mmHg | 5.2 | ( | 1.6 | 8.7 | ) |  | 4.0 | ( | 0.5 | 7.5 | ) |  | C | 0.130 | 0.099 |
| beat/min | -50mmHg | 17.7 | ( | 14.1 | 21.4 | ) |  | 11.7 | ( | 8.1 | 15.3 | ) |  | L | **< 0.001** | 0.859 |
|  |  |  |  |  |  |  |  |  |  |  |  |  |  | C*L | **0.015** | 0.261 |
| ΔSV | -30mmHg | -10.9 | ( | -15.5 | -6.2 | ) |  | -7.8 | ( | -12.4 | -3.2 | ) |  | C | **0.041** | 0.162 |
| ml | -50mmHg | -24.2 | ( | -29.1 | -19.2 | ) |  | -14.9 | ( | -19.6 | -10.1 | ) |  | L | **< 0.001** | 0.616 |
|  |  |  |  |  |  |  |  |  |  |  |  |  |  | C*L | 0.076 | 0.132 |
| ΔCO | -30mmHg | -0.3 | ( | -0.6 | -0.1 | ) |  | -0.2 | ( | -0.5 | 0.0 | ) |  | C | 0.161 | 0.078 |
| L/min | -50mmHg | -0.6 | ( | -0.9 | -0.4 | ) |  | -0.3 | ( | -0.6 | -0.1 | ) |  | L | 0.062 | 0.139 |
|  |  |  |  |  |  |  |  |  |  |  |  |  |  | C*L | 0.353 | 0.036 |
| ΔSVC | -30mmHg | -5.2 | ( | -8.8 | -1.6 | ) |  | -2.0 | ( | -5.7 | 1.6 | ) |  | C | 0.064 | 0.131 |
| ml/min/mmHg | -50mmHg | -7.9 | ( | -11.8 | -4.0 | ) |  | -2.8 | ( | -6.5 | 1.0 | ) |  | L | 0.268 | 0.052 |
|  |  |  |  |  |  |  |  |  |  |  |  |  |  | C*L | 0.524 | 0.017 |
| ΔCBV mean | -30mmHg | -2.8 | ( | -5.8 | 0.1 | ) |  | -2.1 | ( | -5.0 | 0.9 | ) |  | C | **0.040** | 0.257 |
| cm/sec | -50mmHg | -6.5 | ( | -9.7 | -3.4 | ) |  | 0.2 | ( | -2.8 | 3.3 | ) |  | L | 0.594 | 0.021 |
|  |  |  |  |  |  |  |  |  |  |  |  |  |  | C*L | **0.038** | 0.276 |
| ΔCBV peak | -30mmHg | -6.2 | ( | -10.3 | -2.1 | ) |  | -4.6 | ( | -8.7 | -0.5 | ) |  | C | 0.039 | 0.198 |
| cm/sec | -50mmHg | -13.5 | ( | -17.9 | -9.1 | ) |  | -4.4 | ( | -8.6 | -0.2 | ) |  | L | 0.051 | 0.189 |
|  |  |  |  |  |  |  |  |  |  |  |  |  |  | C*L | **0.039** | 0.209 |
| ΔCBV min | -30mmHg | -0.1 | ( | -2.9 | 2.7 | ) |  | 0.5 | ( | -2.3 | 3.3 | ) |  | C | 0.283 | 0.099 |
| cm/sec | -50mmHg | 0.9 | ( | -2.1 | 4.0 | ) |  | 4.0 | ( | 1.1 | 6.9 | ) |  | L | 0.086 | 0.250 |
|  |  |  |  |  |  |  |  |  |  |  |  |  |  | C*L | 0.316 | 0.093 |
| ΔCVCi | -30mmHg | -0.05 | ( | -0.11 | 0.00 | ) |  | -0.01 | ( | -0.06 | 0.05 | ) |  | C | 0.014 | 0.342 |
| cm/sec/mmHg | -50mmHg | -0.08 | ( | -0.14 | -0.02 | ) |  | 0.04 | ( | -0.02 | 0.10 | ) |  | L | 0.671 | 0.013 |
|  |  |  |  |  |  |  |  |  |  |  |  |  |  | C*L | 0.169 | 0.128 |
| ΔEtCO_2_ | -30mmHg | -1.5 | ( | -2.7 | -0.2 | ) |  | -0.9 | ( | -2.2 | 0.3 | ) |  | C | 0.724 | 0.005 |
| % | -50mmHg | -2.2 | ( | -3.5 | -1.0 | ) |  | -2.1 | ( | -3.4 | -0.9 | ) |  | L | **< 0.001** | 0.617 |
|  |  |  |  |  |  |  |  |  |  |  |  |  |  | C*L | 0.245 | 0.064 |

Data are estimated marginal means (EMM) and 95% confidence intervals (CI). Abbreviations are same as Supplemental table 1.
